# CD30 Expression Reveals that Culture Adaptation of Human Embryonic Stem Cells Can Occur Through Differing Routes

**DOI:** 10.1002/stem.41

**Published:** 2009-05

**Authors:** Neil J Harrison, James Barnes, Mark Jones, Duncan Baker, Paul J Gokhale, Peter W Andrews

**Affiliations:** a Centre for Stem Cell Biology, University of Sheffield, Western BankSheffield, United Kingdom; b Department of Biomedical Science, University of Sheffield, Western BankSheffield, United Kingdom; c North Trent Clinical Cytogenetics Service, Sheffield Children's Trust, Western BankSheffield, United Kingdom

**Keywords:** Human embryonic stem cells, CD30, Apoptosis, Chromosomal aberrations, Embryonal carcinoma, Culture adaptation

## Abstract

Human embryonic stem cells undergo adaptive changes that can increase their growth capacity upon prolonged culture in vitro. This is frequently associated with nonrandom karyotypic changes, commonly involving amplification of genetic material from chromosomes 12, 17, and X. A recent study suggested that the karyotypically abnormal cells can be identified by their expression of CD30, which confers resistance to apoptosis. We have now investigated CD30 expression and apoptosis in karyotypically normal and abnormal sublines of the human ES cell line, H7, but our results were contrary to those previously observed. In this cell line, CD30 expression did not segregate the normal and abnormal cells, and abnormal cells were not protected from apoptosis. These data suggest that culture adaptation can occur through a variety of mechanisms.

## INTRODUCTION

Human embryonic stem cells (hESCs) are derived from the inner cell mass of the blastocyst stage embryo and have pluripotent potential [1]. The possibilities these cells afford in regenerative medicine has generated much excitement, but any therapeutic benefits will require controlled hESC maintenance for extended periods in vitro. During growth in culture, however, hESCs have been shown to acquire genetic changes that can increase their growth capacity, a process we have described as “culture adaptation” [2–4]. The increased growth capacity of the culture adapted hESCs must result from a change in their basic behaviors, and understanding adaptation may thus reveal genes important in self-renewal, differentiation, and apoptosis. Changes in such cellular behaviors must also occur during tumorigenesis, and similar karyotypic changes have been observed in culture adapted hESCs and embryonal carcinoma (EC) cells, their malignant counterparts, and the stem cells of testicular germ cell tumors (TGCTs) [4]. Hence, identifying the abnormal stem cells and understanding their growth advantages may have important implications for both stem cell and cancer research.

A report suggested that CD30 is expressed on transformed, karyotypically abnormal hESC but not on normal hESC [5], providing a cell surface antigen indicative of genetic change. CD30 is a member of the tumor necrosis factor (TNF) receptor superfamily, and is expressed on a limited number of cell types which include EC cells [6,7]. CD30 overexpression in Hodgkin–Reed-Sternberg cells has been shown to activate transcription factor nuclear factor kappa-light-chain-enhancer of activated B cells (NF-κB) [8,9], which can promote cell survival through the upregulation of antiapoptotic genes or downregulation of proapoptotic genes [10]. Protection of abnormal cells against apoptosis could provide them with a relative growth advantage over their normal counterparts, allowing them to overtake a culture. Indeed, Herszfeld et al. [5] demonstrated that in a mosaic population of normal and abnormal hESCs, CD30 expression correlated with decreased apoptosis, providing the selection necessary for the karyotypically abnormal CD30-positive hESCs to dominate. Similarly, a more recent study has also shown decreased cell death in karyotypically abnormal hESC [11], suggesting a possible conservation in the mechanism for culture adaptation.

In Sheffield, two karyotypically abnormal sublines of H7 have developed over prolonged culture [2,3], and we investigated whether CD30 expression and apoptotic protection were associated with genetic abnormality in these cell lines. We report that CD30 expression and increased cell survival do not always correlate with genetic aberration, demonstrating that hESCs can adapt to their culture conditions in a variety of ways.

## MATERIALS AND METHODS

### Cell Culture

The H7 hESC line was obtained from Dr. James Thomson, University of Wisconsin, and was maintained and passaged as previously described on mitomycin C-inactivated mouse embryo fibroblasts, in knock-out Dulbecco's modified Eagle's medium (Invitrogen, Carlsbad, CA, http://www.invitrogen.com), supplemented with 10% Serum Replacement (Invitrogen) and 4 ng/ml basic fibroblast growth factor [1,2]. Cells were harvested for passaging by scraping after brief incubation with a solution of 1% collagenase type IV (Invitrogen) in basal medium. Sublines of H7 (H7.s6, H7.s9, and H7.s14) were established in Sheffield. NTERA2 and 2102Ep cells were maintained and passaged as previously described [12,13]. The NTERA2-mCherry line was created using the pCAG-mCherry vector (Invitrogen), with transfection and clone selection methodology as previously described [14].

### Cytogenetic Analyses of hESC

The karyotypic analysis was performed using standard G-banding techniques. Cells cultured in a T25 flask were treated with 0.1 μg/ml Colcemid (Invitrogen) for up to 4 hours, followed by dissociation with trypsin/versene. The cells were pelleted via centrifugation, resuspended in prewarmed 0.0375 M KCl hypotonic solution, and incubated for 10 minutes. Following a further centrifugation step, cells were resuspended in fixative (methanol:acetic acid 3:1). Metaphase spreads were prepared on glass microscope slides and G-banded by brief exposure to trypsin and stained with 4:1 Gurr's/Leishmann's. Between 20 and 30 metaphase spreads were karyotyped each time the cells were analyzed.

Cells were analyzed for trisomy 17 by fluorescent in situ hybridization (FISH) following flow cytometric sorting based on CD30 expression. Collected cells were fixed on glass microscope slides as described previously. Studies were performed using the Qbiogene (Kreatech Biotechnology B.V, Amsterdam, The Netherlands) dual color iso17q probes specific for the genes p53 at 17p13 and myeloperoxidase at 17q23. All hybridizations were performed following the manufacturers instructions. Slides were analyzed on a fluorescent microscope with appropriate filters and software (Cytovision 3.6; Applied Imaging Corporation, San Jose, CA). The signal pattern in 300 interphase cells was examined for each preparation.

### Analyses of Apoptotic Response

#### Assessment of Apoptosis by Annexin V Binding

Cells were cultured to 60%–70% confluency, and treated with either TNF-α (0-400 ng/ml, Sigma) for 24 hours, or Staurosporine (0-1 μg Sigma) for 15 hours, to induce apoptosis. Attached cells were collected following trypsinization, and floating cells were collected from the growth media. The pooled cells were assayed for apoptosis by Annexin V binding. Briefly, the cells were washed once with Dulbecco's phosphate buffered saline (PBS), once with Annexin V binding buffer (10 mM HEPES, 140 mM NaCl; 2.5 mM CaCl_2_, pH 7.4) and then 10^5^ cells incubated with 20 μl recombinant human Annexin V: Fluorescein isothiocyanate (FITC) (Invitrogen) for 20 minutes at room temperature. Propidium Iodide (PI) solution (Sigma) was then added to the incubation mix at final concentration of 0.01 mg/ml, before samples were analyzed on the CyAnADP O2 (Dako, Glostrup, Denmark, http://www.dako.com).

#### Assessment of Apoptosis by Caspase-3 Activation

Cells were cultured to 60%–70% confluency, and the levels of caspase-3 activation measured following staurosporine treatment. Cells were collected as for the Annexin V assay, then washed once with PBS before fixation with 4% paraformaldehyde (PFA, 15 min at room temperature). Cells were permeabilized using 0.3% Triton-X (10 min, analaR), washed once with PBS, once with PBS supplemented with 5% fetal calf serum (Fluorescence-activated cell sorting [FACS] buffer), and then incubated with a rabbit polyclonal to active Caspase-3 (1:200, Abcam, Cambridge, U.K., http://www.abcam.com) for 45 min at 4°C, with occasional shaking. Cells were then washed three times with FACS buffer, before similar incubation with an Alexa Flour-555 donkey anti-rabbit IgG (H+L) (1:100; Invitrogen). Cells were again washed three times with FACS buffer and analyzed on the CyAnADP O2 (Dako).

#### Western Blotting for Caspase-8 Activation

The cells were treated with TNF-α (400 ng/ml) as previously described and caspase-8 cleavage was tested. Briefly, samples were lysed using Chaps cell extract buffer (50 mM Pipes/HCl, 2 mM EDTA, 0.1% Chaps, 20 μg/ml Leupeptin, 10 μg/ml Pepstatin A, 10 μg/ml aprotonin, 5 mM dithiothreitol) followed by resuspension, freezing, thawing (three times), and centrifugation at 1,400 rpm to pellet cell debris. SDS buffer was added to the supernatants, which were then boiled and the protein concentration determined using the Bio-Rad protein assay (Bio-Rad, Hercules, CA, http://www.bio-rad.com), according to manufacturers' instruction. Samples were then diluted to equal protein concentrations prior to running on an SDS-PAGE gel. Proteins were electroblotted onto nitrocellulose membranes with 0.45 μm pores, and the membranes were blocked by incubation for 1 hour with blocking buffer (1× tris buffered saline [TBS], 0.1% Tween-20, 5% [w/v] nonfat dry milk). The membrane was washed three times with wash buffer (1× TBS, 0.1% Tween-20), before incubation with cleaved-caspase-8 primary antibody (cell signaling technology, diluted in blocking buffer) overnight at 4°C with shaking. Membranes were then washed again three times with wash buffer, and incubated with horseradish peroxidase-conjugated secondary antibody (diluted in blocking buffer) for 1 hour at room temperature. Blots were developed with the enhanced chemiluminescence detection system (ECL; Pierce Chemical, Rockford, IL, http://www.piercenet.com).

### Analyses of CD30 Expression

#### Flow Cytometry, Cell Sorting, and Immunoflourescence

Cells were suspended in FACS buffer following trypsinization, and 10^7^ cells taken for staining. Cells were incubated with anti-CD30 primary antibody (mouse monoclonal anti-human CD30, clone Ber-H2, 1:50, Dako) for 45 min at 4°C with occasional shaking, and then washed three times with FACS buffer. Following the final wash, the cells were resuspended in FACS buffer, and incubated with either a FITC-conjugated Goat anti-mouse secondary antibody (1:100; Caltag) or an allophycocyanin-conjugated Goat anti-mouse secondary antibody (1:100; Molecular probes, Eugene, OR, http://probes.invitrogen.com) for 45 min at 4°C with occasional shaking. Cells were washed three times with FACS buffer again, and analyzed on either the CyAnADP O2 (Dako) flow cytometer or the MoFlo (Dako). For a negative control, hESC were stained with secondary antibody only, as described earlier. For a positive control EC cell lines NTERA2 and 2102Ep, known to express CD30, were analyzed as described above. In addition, 10^6^ NTERA2 cells, which constitutively expressed the mCherry flourescent protein, were mixed with 10^6^ H7.s6 and also H7.s14 cells, and the cells assayed as described. For cytogenetic and clonogenic assays, the stained cells were sorted according to CD30 expression. For the clonogenic studies, CD30-positive and CD30-negative populations were seeded at densities of 1000-6000 cells/well into 6-well plates containing mitomycin C-inactivated mouse embryo fibroblasts. For immunofluorescent analyses, colonies were fixed 7-10 days postseeding using 4% PFA as previously described, and stained using anti-TRA-1-60 primary antibody (1 hour, 4°C; produced in-house) followed by FITC-conjugated secondary antibody (1 hour, 4°C; Caltag), and imaged using the IN Cell Analyser 1,000 (Amersham Biosciences, Piscataway, NJ, http://www.amersham.com). Secondary antibody only was used as negative control. Cell nuclei were visualized using Hoescht 33,342 staining (10 μg/ml; Sigma). For cloning efficiency, fixed colonies were visualized and counted following staining with crystal violet (1 hour; Sigma).

#### Apoptotic Analyses

Cells were treated with or without 0.025 μM Staurosporine (15-hour treatment) and harvested for flow cytometric as described earlier. After staining the cells for CD30 expression, as described, they were washed once with Annexin binding buffer, and 10^5^ cells taken and stained with 20 μl recombinant human Annexin V: APC (Invitrogen), using the procedure previously described.

## RESULTS

### Karyotyping of HESC Lines

**Figure 1:**
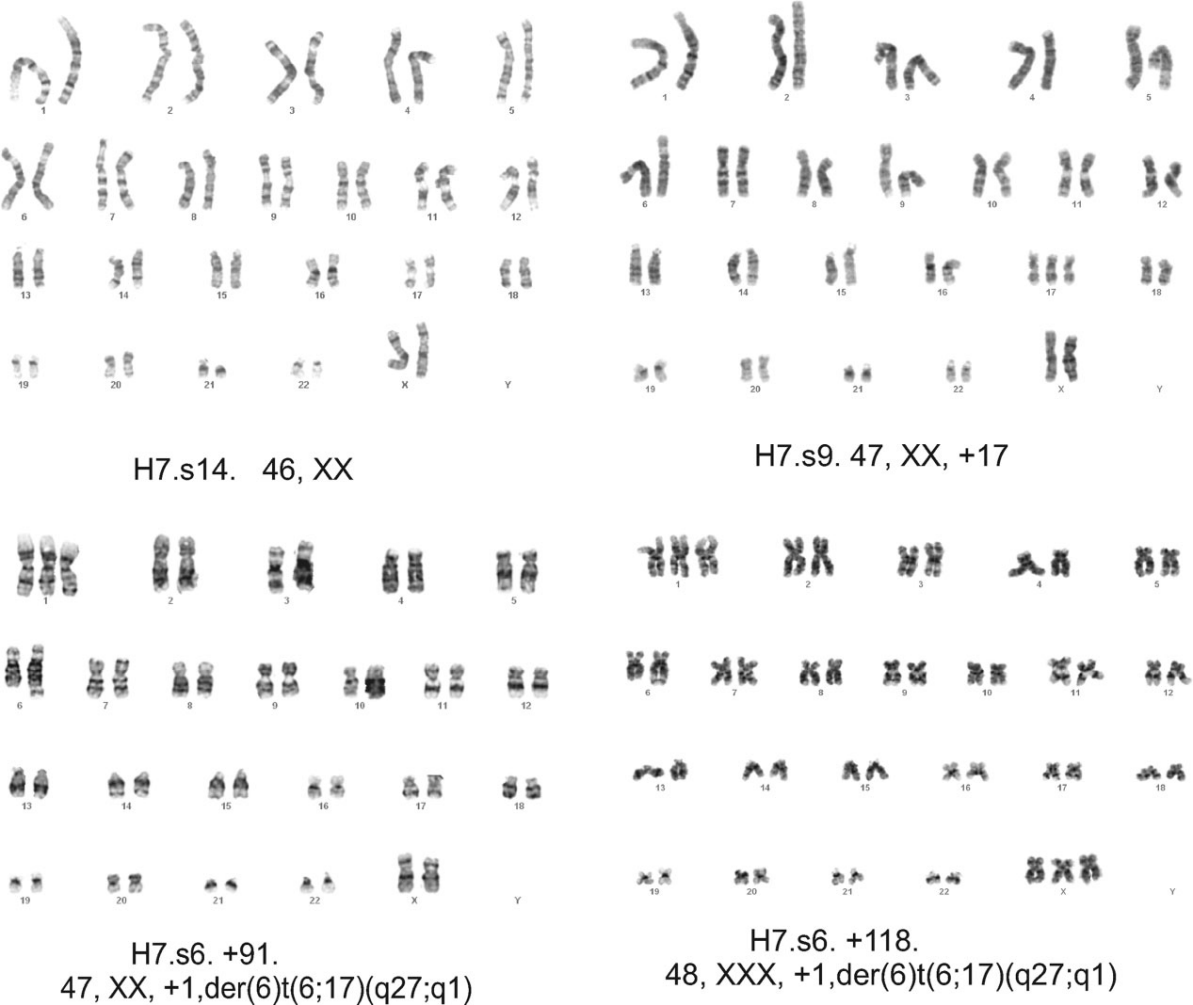
Karyotypic of analyses of the H7 sublines. The ideograms show the karyotypes of the H7.s14, H7.s9, and H7.s6 lines at the passage number stated.

The 3 hESC sublines used were denoted as H7.s14, H7.s6, and H7.s9, and the ideograms demonstrating their karyotype are shown in Figure 1. The H7.s14 cells maintained a normal diploid 46, XX karyotype throughout the study, whereas the H7.s6 cells had already adapted to culture (47, XX, +1, der(6)t(6, 17)(q27;q1) [3] and also acquired an extra copy of chromosome X through the course of the study (48, XXX, +1, der(6)t(6, 17)(q27;q1)). H7.s6 and H7.s14 were cultured for 30-35 passages during this study. The H7.s9 line began as a mosaic population containing diploid cells and also cells trisomic for chromosome 17. By the time the cells had undergone 45 passages, the abnormal, adapted cells had overtaken the normal cells so the H7.s9 culture was entirely 47, XX +17, and this karyotype was unchanged during a further 30 passages. For the purpose of this study, when hESC are termed ‘normal’, this refers only to their karyotypic status.

### Analyses of Apoptotic Pathways

**Figure 2:**
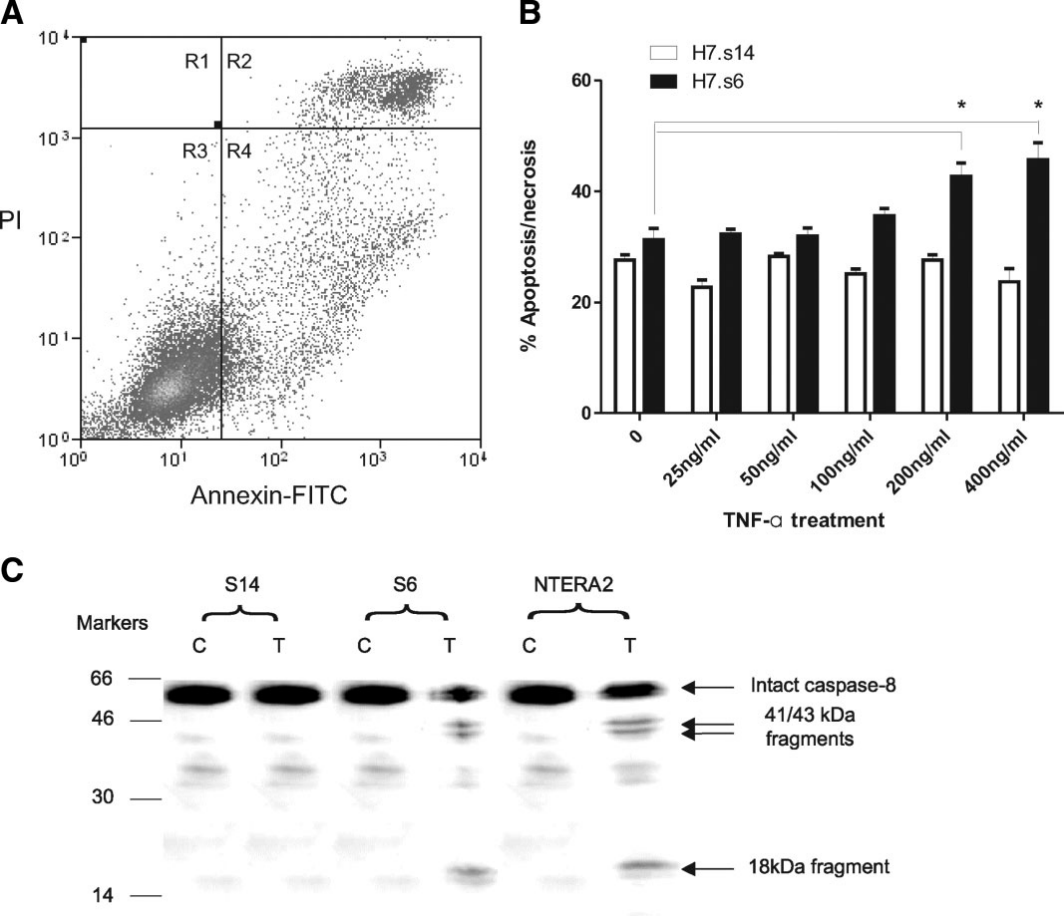
Analyses of the extrinsic apoptotic pathway in normal and culture adapted human embryonic stem cells. **(A):** Representative histogram showing Annexin V binding in H7.s6 cells, with the apoptotic/necrotic population a sum of R2, R3, and R4. **(B):** Measurement of apoptosis/necrosis in normal H7.s14 and culture adapted H7.s6 cells following TNF-α treatment, as measured by Annexin V binding. **(C):** Western blot for cleaved caspase-8 in H7.s14, H7.s6, and NTERA2 cells. Abbreviations: C, control treated; T, TNF-α treated (400 ng/ml); TNF-α, tumor necrosis factor-alpha.

The extrinsic (death receptor dependant) and intrinsic (mitochondrial dependant) apoptotic pathways were studied in the H7.s14 and H7.s6 cells, to determine any differences between this normal/adapted pair. The addition of TNF-α should activate the extrinsic apoptotic pathway [15], yet the H7.s14 subline seemed unresponsive to this compound, as assessed by Annexin V binding. The H7.s6 subline however, showed significant increases in apoptosis/necrosis when treated with 200 and 400 ng/ml TNF-α (Student's *t* test *p* ≤ .05, *n* ≥ 3; Fig. 2A, B). Activation of caspase-8 is downstream of the death receptors, and cleavage of this enzyme was studied by Western blotting (Fig. 2C). As expected, cleavage of caspase-8 was only seen in the H7.s6 cells. To test the proposed similarity between culture adapted hESC and EC cells, caspase-8 cleavage was assayed in NTERA2 cells, which also showed activation of this enzyme following TNF-α treatment.

**Figure 3:**
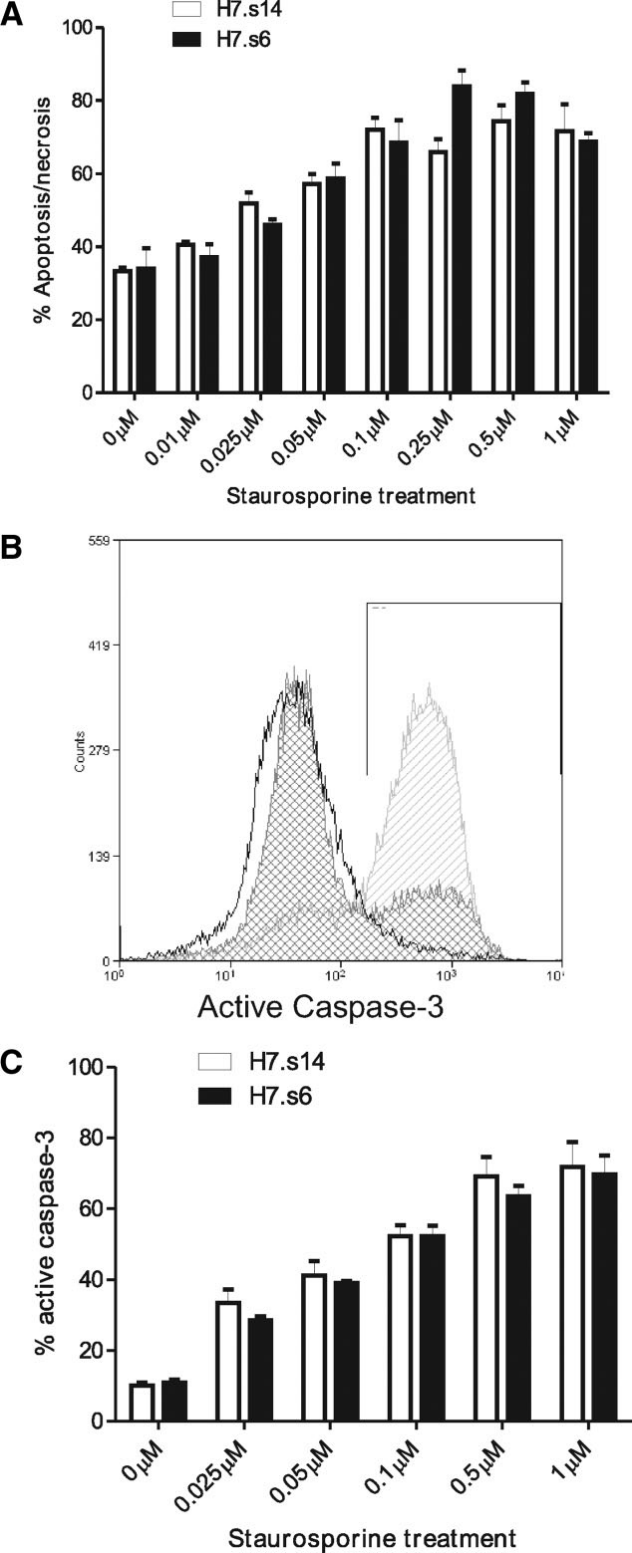
Analyses of the intrinsic apoptotic pathway in normal and culture adapted human embryonic stem cells (hESC). **(A):** Measurement of apoptosis/necrosis in normal H7.s14 and culture adapted H7.s6 cells following staurosporine treatment, as measured by Annexin V binding. **(B):** Sample histogram showing caspase-3 activation in untreated hESC (no fill), and following treatment with 0.025 μM (dark gray, checked) and 1 μM (light gray, striped) staurosporine. **(C):** Measurement of apoptosis in H7.s14 and H7.s6 cells following staurosporine treatment, as measured by caspase-3 activation.

The intrinsic apoptotic pathway was interrogated by addition of staurosporine (0.05-1 μM) to the cells, which causes rapid activation of this mitochrondrial pathway [16]. Cell death was measured by Annexin V binding, and also caspase-3 activation, in the H7.s6 and H7.s14 sublines. The activation of caspase-3 was chosen as a secondary measure of apoptosis since this has been recognized as the crucial executioner caspase [17]. Both lines displayed significant increases in apoptosis when compared with the control treatment at concentrations ≤0.025 μM (Student's *t* test, *p* ≤ .05, *n* ≥ 3), suggesting that the intrinsic pathway is active in these cells. However, based on Annexin V binding and caspase-3 activation, no significant variation was seen in apoptotic response between the normal and abnormal sublines across the range of staurosporine concentrations tested (Student's *t* test, *p* ≤ .05, *n* ≥ 3; Fig. 3). The levels of hESC apoptosis/necrosis indicated by Annexin V binding were typically higher than the levels of apoptosis predicted by caspase-3 activation, most particularly in the untreated cells (apoptosis/necrosis at ∼30% as measured by Annexin V binding, but apoptosis only at ∼10% by caspase-3 activation), suggesting that hESC death may occur through a pathway which can bypass caspase-3.

### Analyses of CD30 Expression in HES and EC Cell Lines

**Figure 4:**
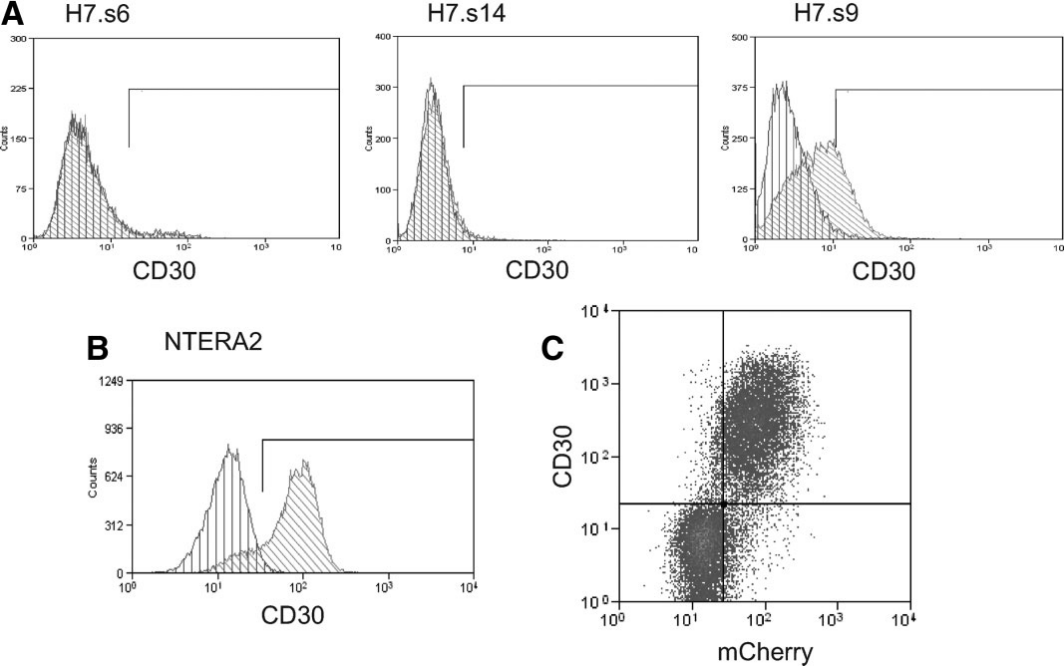
CD30 expression on embryonal carcinoma, normal and culture adapted human embryonic stem cell (hESC) lines. **(A):** CD30 expression in the H7.s14, H7.s6, and H7.s9 hESC lines. The negative control (cells stained with secondary antibody only, vertical lines) and cells stained for CD30 (diagonal lines) are represented in the histograms. **(B):** Sample histogram showing CD30 expression in the NTERA2 embryonal carcinoma (EC) line, with control and stained cells depicted as in previous. **(C):** Sample histogram showing the CD30 binding profile in a mixed population of mCherry NTERA2 cells and H7.s6 cells, demonstrating expression of this antigen is only observed in the fluorescent EC line.

Flow cytometric analyses showed that neither the H7.s14 nor the H7.s6 lines displayed the CD30 antigen (Fig. 4A), despite the H7.s6 cells undergoing further karyotypic change during the course of the study. As a positive control CD30 expression was also tested on NTERA2 and 2102Ep EC cells [7], which displayed obvious expression of CD30 (Fig. 4B). Further verification of the H7.s6 and H7.s14 staining results was provided by mixing equal numbers of fluorescent NTERA2 cells (constitutively expressing an mCherry construct) with cells from each H7 subline. Here, CD30-positive staining was only observed in those cells expressing mCherry (the NTERA2 cells), and not the hESC (Fig. 4C).

**Figure 5:**
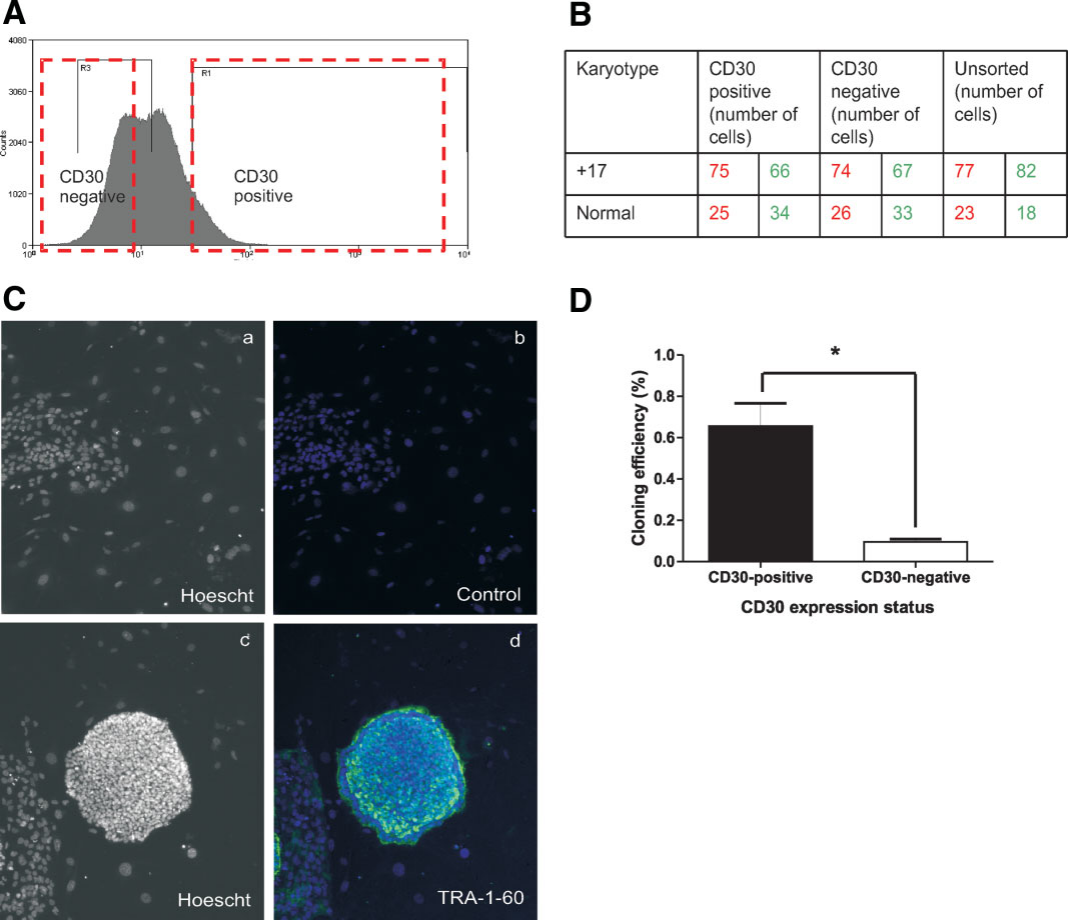
Fluorescent sorting of H7.s9 cells based on CD30 expression. **(A):** Cells sorted for cytogenetic and clonogenic analyses, with the boxed areas representing the CD30 positive and negative fractions taken. **(B):** Number of normal and abnormal cells in the fractions analyzed, assessed by fluorescent in situ hybridization. The data shown is from two separate assays, with data from the first assay in red, and the second assay in green. **(C):** TRA-1-60 expression in hESC derived from CD30-positive H7.s9 cells: Hoescht only (Ca, Cc), FITC-conjugated secondary antibody-only control and Hoescht (Cb), TRA-160 and Hoescht (Cd) (original magnification, ×4). **(D):** Cloning efficiency of the CD30-positive and CD30-negative populations, the CD30-positive population has a significantly higher cloning efficiency (Student's *t* test, *p* ≤ .05, *n* ≥ 3).

The expression of CD30 was also tested in another subline of H7 (H7.s9), initially at stages when the entire culture was karyotypically abnormal (47, XX +17), and a population of CD30-positive cells (∼30%) (Fig. 4A) was observed in these cultures throughout the study. Fluorescent staining for CD30 was also performed on earlier passage H7.s9, while the culture was still mosaic, and this population also contained ∼30% CD30-positive cells. The mosaic H7.s9 cells were sorted as CD30-positive or negative from two consecutive passages and analyzed by FISH, in addition to a sample of unstained cells. Analyses for trisomy at chromosome 17 revealed that abnormal cells could not be segregated from normal cells based on CD30 expression (Fig. 5A, B). As CD30 expression was relatively low, clonogenic assays were performed to confirm that the CD30-positive cells were true hESC, and could reform clonal colonies. Here, colonies were observed 7-10 days post seeding in which TRA-1-60 was expressed, suggesting the presence of undifferentiated hESC (Fig. 5C). In addition, the cloning efficiency of both the CD30-positive and CD30-negative cells was measured. Both populations exhibited clonogenic capacity, yet the CD30-positive cells had a significantly higher cloning efficiency (Student's *t* test, *p* ≤ .05, *n* ≥ 3; Fig. 5D).

### CD30 Expression and Cell Survival

**Figure 6:**
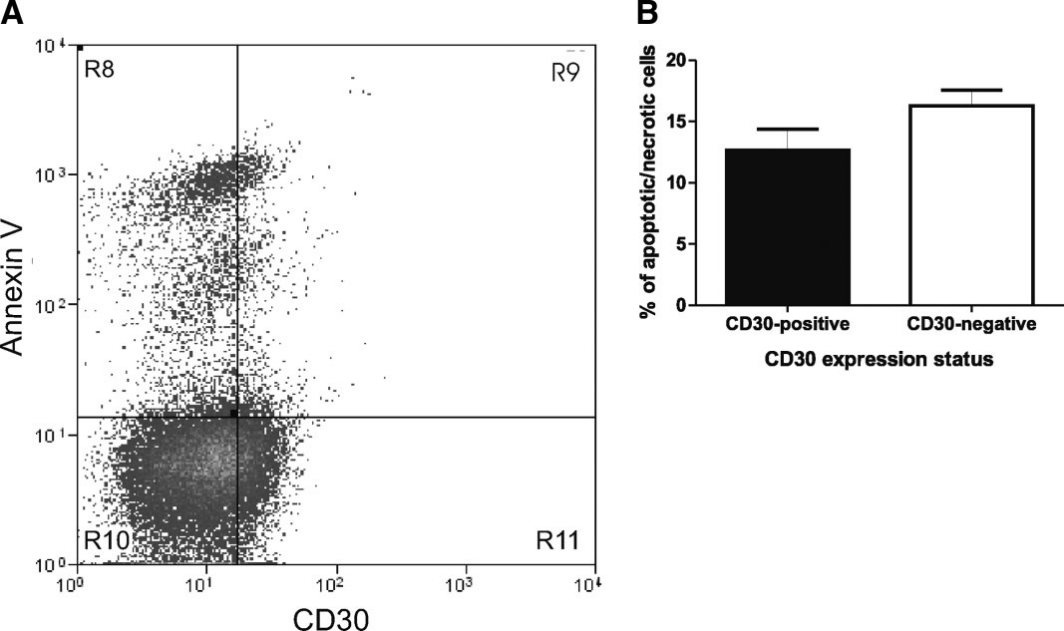
Analyses of apoptosis in CD30-positive and CD30-negative cells. **(A):** Representative histogram showing dual staining of H7.s9 human embryonic stem cells (hESC) with AnnexinV:Cy5 and CD30:FITC. **(B):** Bar chart plotting the percentage of apoptotic/necrotic cells in the CD30-positive and CD30-negative populations, revealing no significant difference (student's *t*-test, *p* < .05, *n* ≥ 3).

The mosaic expression of CD30 in the H7.s9 subline was exploited to test whether the expression of this protein protected cells against apoptosis under normal culture conditions. H7.s9 cells were dual stained for CD30 expression and Annexin V binding, revealing spontaneous apoptosis/necrosis in both the CD30-positive and negative populations (Fig. 6A). The proportion of apoptotic/necrotic cells was not significantly different between the CD30-positive and negative cells (Student's *t* test, *p* ≤ .05, *n* ≥ 3; Fig. 6B), revealing that CD30 expression does not appear to protect against spontaneous apoptosis in this subline under our laboratory conditions. In addition, comparing the levels of cell death in the untreated H7.s9 to the untreated H7.s14 line (30.3% ± 4.3% Annexin V binding and 14.68% ± 2.85% caspase-3 activation in H7.s9) revealed no significant difference between this normal/abnormal pair. To determine whether CD30 expression protected against induced apoptosis, H7.s9 cells were treated with 0.025 μM staurosporine and CD30 expression and Annexin V binding similarly measured. In the treated cells levels of apoptosis were similar to those observed in the H7.s14 and H7.s6 lines (40%–50%), and no CD30 expression was observed (data not shown). It seems likely that the CD30-positive population has been preferentially depleted, suggesting these cells are not protected from death induced by staurosporine. However, it should be noted that staurosporine is a highly unspecific protein kinase inhibitor, and the deleterious impact it has on the CD30-positive population may not relate solely to apoptotic sensitivity.

## DISCUSSION

In contrast to previous reports, our data have found that CD30 expression does not definitively denote karyotypically abnormal cells, and that its expression does not always correlate with reduced cell death. Indeed, we have shown that the adaptation of hESC to culture does not always result from an increased capacity for survival, and in fact some abnormal lines may be more sensitive to particular apoptotic stimuli. These data suggest that culture adaptation can occur through more than one pathway, and as such there is likely to be variation between different culture adapted lines.

CD30 was not expressed in either the normal H7.s14 subline or the long established culture adapted H7.s6 subline, but was observed on cells of a further abnormal subline (H7.s9). Sorting of hESC cells based on CD30 expression was unable to segregate normal and abnormal cells, demonstrating that in this particular subline CD30 does not provide an indicator of karyotypic change. Also, the apoptotic data from the H7.s9 line showed no correlation between CD30 expression and increased cell survival. There was no obvious advantage for the CD30-positive cells under normal culture conditions, since the levels of CD30 expression not show a sustained increase during the study, and never exceeded 50% of the culture. On the other hand, the CD30-positive cells did show an increased cloning efficiency (approximately sevenfold) compared to the CD30-negative cells. However, growth conditions during clonogenic assays are markedly different from those during normal hESC culture, and it is possible that CD30 might have a specific effect on the regrowth of singlet cells. In this regard it notable that CD30 has recently been reported as marker of undifferentiated cells [18], such that in certain hESC lines it may act akin to, for example, SSEA3, in recognizing a pluripotent, clonogenic population rather than abnormal variants.

In addition to cell surface marker expression, this study also focused on the apoptotic pathways of the H7.s14 and H7.s6 cell lines. An increase in resistance to apoptosis in the culture adapted H7.s6 cells would provide an elegant explanation for their increased growth capacity, yet when the extrinsic and intrinsic apoptotic pathways were stimulated in both cell lines, no decrease in apoptosis/necrosis was observed in the H7.s6 when compared with the normal H7.s14 cells. In fact, when the extrinsic pathway is further stimulated with high concentrations of TNF-α, the H7.s6 cells actually appear more prone to death through this pathway. The activation of this death receptor pathway is also observed in the EC NTERA2 cell line, and supports hESC culture adaptation as a paradigm for TGCT development [19]. The increased sensitivity to apoptosis in the H7.s6 and NTERA2 cells may result from an escape from cell cycle regulation, fitting a rapidly proliferating malignant phenotype. If culture adaptation is considered a model for germ cell tumor development then one might expect to discover the expression of CD30 in the abnormal hESC, as this marker is present in almost all EC lines. However, the time at which EC lines acquire CD30 expression is unknown, and it may mark a late stage in tumor progression, whereas the H7.s6 line may be more closely related to an earlier stage. Indeed, the gross karyotypic abnormalities observed in EC cells are not present in the H7.s6 cells, and the expression of CD30 may relate to a genetic change this line will later acquire.

Aside from the comparison between normal and abnormal hESC lines, the data presented here may also offer insight into hESC cell death. Annexin V binding was utilized as a measure of apoptosis/necrosis, and caspase-3 activation as a measure of apoptotic cell death, yet these 2 methodologies gave differing results. The spontaneous apoptosis/necrosis predicted by Annexin V binding was in the region of 30% for all cell lines, yet the proportion of caspase-3 activation observed was closer to 10%. Although the levels of Annexin V binding may appear high, they are similar to those reported in previous studies [20–22], and indeed up to 53% Annexin V binding has been observed in hESC cultures maintained by Pyle et al. [23]. Considering the population doubling time of hESC is commonly estimated as upwards of 30 hours [3,23–25], yet the cycle time of these cells may be as short as 15-16 hours [26], a sizeable fraction of the population must not survive the rigors of in vitro existence and it is unlikely that the Annexin V binding assay is over-reporting cell death. As such, it raises the possibility that hESC death is mediated through a pathway independent of caspase-3, classically considering the central player in the apoptotic response. Nonapoptotic cell death has been reported [27], as has a caspase-2-dependent apoptotic program capable of bypassing caspase-3 [28], and it is tempting to speculate that such processes are responsible for the cell death observed during routine culture of hESC.

## SUMMARY

This study reveals variations in behavior that can occur during hESC culture adaptation. The differences observed in apoptotic sensitivity between our abnormal cell lines and those of Herszfeld et al. [5] provide evidence that culture adaptation can occur through multiple routes, and that these may be related to different culture conditions and/or epi/genetic changes. Some mutations may protect against apoptosis, yet others may reflect changes in, for example, the propensity for self-renewal. Hence, we propose culture adaptation as a potentially multifaceted process, affording the opportunity to study a number of cell behaviors key to hESC maintenance, and also act as a paradigm for tumor development.

## DISCLOSURE OF POTENTIAL CONFLICTS OF INTEREST

P.W. Andrews served as an officer or member of the board of Axordia.
